# Effects of dance and music training on the plasticity of the core empathy network: intra- and inter-network connectivity

**DOI:** 10.7717/peerj.21811

**Published:** 2026-10-22

**Authors:** Yuling Liu, Hui He, Gujing Li, Peng Sun, Shenghuan Deng, Yayun Liu, Benjamin Klugah-Brown, Yuanyuan Yu, Mingjun Duan, María Luisa Bringas-Vega, Roberto Rodríguez-Labrada, Jing Lu, Frank Pollick, Lixue Yin, Tuomeiciren Heyang, Dezhong Yao, Cheng Luo

**Affiliations:** 1 School of Marxism, University of Electronic Science and Technology of China, Chengdu, China; 2 i-Large Model Innovation Lab of Ideological and Political Science, University of Electronic Science and Technology of China, Chengdu, China; 3 School of Life Science and Technology, The Clinical Hospital of Chengdu Brain Science Institute, MOE Key Lab for Neuroinformation, University of Electronic Science and Technology of China, Chengdu, China; 4 The Clinical Hospital of Chengdu Brain Science Institute, Department of Psychiatry, Chengdu Mental Health Centre, The Fourth People’s Hospital of Chengdu, Chengdu, China; 5 Cuban Neuroscience Center, La Habana, Cuba; 6 School of Psychology and Neuroscience, University of Glasgow, Glasgow, United Kingdom; 7 Sichuan Provincial People’s Hospital, Chengdu, China; 8 College of Chinese and ASEAN Arts, Chengdu University, Chengdu, China; 9 China-Cuba Belt and Road Joint Laboratory on Neurotechnology and Brain-Apparatus Communication, University of Electronic Science and Technology of China, Chengdu, China

**Keywords:** Dance training, Music training, fMRI, Empathy, Mirror neuron system

## Abstract

**Background:**

Dance and music are two of the oldest and most universal forms of artistic expression, each requiring extensive training. Prolonged training is known to induce neuroplastic changes and enhance socio-emotional abilities such as empathy. The mirror neuron system (MNS) is associated with empathy; however, few studies systematically explored how dance and music training modulate the core empathy network, including subcortical regions within the limbic MNS.

**Method:**

Resting-state functional magnetic resonance imaging (fMRI) data were obtained from 25 dancers, 24 musicians and 39 demographically matched control group (both groups are healthy). Core regions within relevant brain networks, along with key subcortical components of limbic MNS, specifically the amygdala and basal ganglia, were defined as regions of interest (ROIs) to build a core empathy network. Graph-theoretical metrics, including within-network degree (WND) and between-network degree (BND), were calculated to evaluate connectivity patterns. These metrics were further correlated with empathy scores.

**Results:**

Both dancers and musicians exhibited altered connectivity within the core empathy network. Dancers showed increased WND and BND between basal ganglia regions and the ventral attention network, whereas musicians demonstrated enhanced WND between the amygdala and the subcortical network. Notably, both groups exhibited reduced WND between the superior frontal gyrus and the sensorimotor network. The neural changes were correlated with Interpersonal Reactivity Index (IRI) empathy subscale scores, indicating training-related modulations in affective and cognitive empathy.

**Conclusion:**

Dance and music training induce both distinct and overlapping patterns of neuroplasticity within the core empathy network that are associated with enhanced empathic ability. The findings support the potential of dance and music as systematic intervention programs.

## Introduction

Dance and music are the two oldest and most common forms of human art and social life. They are not only the mediums of cultural expression and emotional communication, but also can contribute positively to cognitive processing and social skills (Elst et al., 2023). Increasingly, evidence demonstrates the ability of long-term dance or music training to enhance the empathy of healthy individuals and improve the emotional regulation of patients with mental illness, thus alleviating clinical symptoms (Koch et al., 2019; Millman et al., 2021; Wu & Lu, 2021). These research results show that dance and music may positively influence the functional organization of the brain’s core empathy network (Yu et al., 2025). It is important to note that the neuroplastic outcomes of such training likely depend on the specific modalities involved. For instance, evidence suggests that dance forms emphasizing interpersonal coordination and rhythmic synchronization tend to foster empathic functions differently than those focused primarily on individual technical execution (Koehne, Schmidt & Dziobek, 2016). Acknowledging these variations is essential for understanding how distinct artistic practices, and their specific training demands, differentially modulate the neural systems underlying emotional resonance and social understanding.

Previous studies have shown that dance and music training can enhance functional connectivity among emotional-related brain regions, such as the insula, anterior cingulate cortex (ACC) and middle temporal gyrus, thus improving empathy (Li et al., 2019). Other evidence indicates that the mirror neuron system (MNS) constitutes a key neural mechanism through which dance and music shape the core empathy network (McGarry & Russo, 2011). The MNS serves as a crucial sensorimotor simulation system, that enables individuals to comprehend the intentions and emotional states of others by mapping the observed actions and expressions onto their own motor representations (Plata-Bello et al., 2023). Current research indicates that MNS activity is significantly modulated by associative sensorimotor learning and specialized training (Heyes & Catmur, 2022). Functionally, the MNS can be divided into two subsystems. The core MNS, located along the dorsal pathway and encompassing the premotor cortex, inferior frontal gyrus, and inferior parietal lobule, supports sensory-action coupling for imitation (Rizzolatti et al., 1996). The limbic MNS, located in the ventral pathway and involving structures like the insula, amygdala, and basal ganglia, plays a central role in emotional mirroring. Recent findings suggest that affective empathy involves the activation of visceromotor pathways in the observer, rather than being limited to somatomotor simulation (Bonini et al., 2022). Given the critical role of the MNS in empathy, researchers have constructed a core empathy network model based on regions within this system (Fan et al., 2011; Shamay-Tsoory, 2011). However, most of these models focus primarily on cortical structures, with relatively little attention paid to subcortical regions (Singer et al., 2004). Importantly, several subcortical areas within the limbic system, particularly the amygdala and basal ganglia, are closely associated with emotional mirroring and serve as key neural substrates underlying empathic processing (Haris et al., 2023).

Therefore, incorporating subcortical structures into the core empathy network is necessary for achieving a more comprehensive characterisation of its neural architecture. Furthermore, although dance and music training have both been identified as effective approaches for enhancing empathy (Li et al., 2019; Wu et al., 2023), their respective effects on the plasticity of the core empathy network, as well as their shared and distinct neuroplastic mechanisms, remain unclear. To address this gap, the present study employed neuroimaging methods to investigate how dance and music training modulate the plasticity of the core empathy network. The research results are expected to deepen our understanding of the neural basis through which artistic training enhances empathy and provide important theoretical inspiration for the intervention and rehabilitation of related mental illnesses.

Recent advancements in magnetic resonance imaging (MRI) have provided a valuable tool for investigating the core empathy network’s characteristics. Functional magnetic resonance imaging (fMRI) studies have identified key regions associated with empathy, including the dorsal anterior cingulate cortex, mid-anterior cingulate cortex, supplementary motor area, and bilateral anterior insula (Fan et al., 2011). However, systematic analyses of the core empathy network from the perspective of brain network topology remain limited. Recent advances in graph-theoretical network analysis have enabled quantitative characterisation of connection patterns among neural nodes, offering a novel perspective for examining how dance and music training influence the plasticity of the core empathy network (Sporns & Betzel, 2016). Within this framework, the brain can be modeled as a complex network composed of nodes and edges, and metrics such as within-module degree (WMD) have been widely used to quantify the strength of intramodular connectivity (Guimerà & Amaral, 2005; Hwang et al., 2017). Building upon this approach, this study introduced two new network metrics, WND and between-network degree (BND), to evaluate the strength of intra-network connections and the efficiency of inter-network information transfer, respectively. These two metrics are expected to deepen our understanding of how dance and music training regulate the information integration and processing in the core empathy network.

In summary, building upon established research frameworks for the cortical empathy core networks (Fan et al., 2011; Shamay-Tsoory, 2011) and dissociable intrinsic connectivity networks (Seeley et al., 2007), as well as the analytical findings from the preceding stages of this study (Li et al., 2026), we further incorporated key subcortical structures of the limbic mirror neuron system—specifically the amygdala and the basal ganglia—as regions of interest (ROIs) to expand upon the core empathy network. Resting-state fMRI data were obtained from dancers, musicians, and a matched control group. We systematically compared the similarities and differences of the neuroplasticity mechanisms underlying how dance and music training enhance empathy by calculating WND and BND within the 41 selected ROIs across the three groups. It is expected that these research results will provide important insights into how dance and music training can optimize the neural foundations of empathy, as well as provide valuable guidance for the intervention and rehabilitation of related mental illnesses.

## Materials and Methods

### Participants

This study involved 25 dancers, 24 musicians, and 39 age-matched and sex-matched healthy controls from the University of Electronic Science and Technology of China and Southwest Minzu University. Participants were recruited and data collection was conducted between May 2022 and July 2022 (see Table 1 for detailed demographic information). Musicians were included in the study because both music and dance training engage the MNS through shared sensorimotor and emotional demands, yet differ in their primary modalities: dance relies on whole-body physical imitation, whereas music emphasizes fine auditory-motor coupling. Participants with irregular training histories were excluded through questionnaire screening. As a result, all dancers and musicians had undergone more than 7 years of formal training, practised for an average of 12 h per week, and had passed professional evaluations by senior dance or music instructors. The dancers primarily specialized in modern dance, while the musicians played a variety of Western classical and traditional Chinese instruments. Dancers were specifically assessed through three tasks, which were designed and evaluated in consultation with professional dance instructors to strictly verify expert status: (1) a flexibility task, which required performing movements such as backbends and splits, combined with a series of technical sequences according to individual flexibility; (2) a mimicry task, in which dancers observed a four-bar sequence demonstrated three times and were asked to recall and reproduce the sequence as accurately as possible; and (3) an improvisation task, requiring the spontaneous creation of a dance to randomly selected music. Musicians were evaluated using two tasks: (1) a continuous performance task, which required the prepared performance of a musical piece lasting 3 min; and (2) a sight-reading task, in which they were asked to read and perform 16 measures of unfamiliar music without prior preparation. Conversely, the control group had no background in professional dance or music training.

**Table 1:** Demographic characteristics of participants. Age, sex distribution, and years of education for the three study groups. *P*-values indicate no significant differences between groups in any demographic category.

Characteristics	Dancers (*n* = 25)	Musicians (*n* = 24)	Controls (*n* = 39)	*p*-value
Age range (years)	17–27	18–24	18–26	–
Mean ± SD (years)	20.04 ± 2.49	19.92 ± 1.44	20.51 ± 1.55	0.396^a^
Sex (Male/Female)	8/17	15/9	19/20	0.182^b^
Education (years)	13.76 ± 2.33	13.83 ± 1.63	14.69 ± 1.44	0.069^a^

**Notes:**

a *p*-values were calculated using one-way analysis of variance (ANOVA).

b *p*-value was calculated using the Chi-square test.

Importantly, while neuroimaging analyses were performed on the full sample (*n* = 88), correlation analyses with empathy scores were conducted on a subset (*n* = 51: 16 dancers, 15 musicians, and 20 controls) who provided complete behavioral scale data. SD, standard deviation.

All participants met the following inclusion criteria: right-handedness, as determined by the Edinburgh Handedness Inventory (Oldfield, 1971); no metallic implants (*e.g*., pacemakers, surgical plates); no contraindications for MRI scanning (*e.g*., claustrophobia); no history of brain injury, developmental disorders, neurological or psychiatric conditions; no chronic conditions such as hypertension or cardiovascular disease; and no history of alcohol or substance abuse.

The study was preregistered with the Chinese Clinical Trial Registry (ChiCTR2200059526) and received approval from the Ethics Committee of the University of Electronic Science and Technology of China (approval number: 1061420210829009). Prior to the experiment, written informed consent was obtained from all participants, and all procedures were conducted in compliance with the Declaration of Helsinki (World Medical Association, 2002).

### Scale measurement of empathy

In this study, participants’ empathic ability was evaluated using the Chinese version of the Interpersonal Reactivity Index (C-IRI), a self-report instrument originally developed by Davis and subsequently translated and adapted by Chinese scholars (Davis, 1980). The reliability and validity of the C-IRI have been rigorously validated through psychometric evaluation (Rong et al., 2010). The scale consists of 28 self-report items rated on a five-point Likert scale (0 = not descriptive of me at all to 4 = very descriptive of me), with higher scores indicating a stronger tendency to be empathetic in interpersonal interactions. The C-IRI comprises four relatively independent subscales: perspective taking (PT), fantasy (FS), empathic concern (EC), and personal distress (PD). Specifically, the PT subscale assesses an individual’s tendency to take on others’ perspectives; the FS subscale evaluates the capacity to imagine or feel the emotions of characters in fictional situations; the EC subscale measures sympathy and concern for individuals in adverse circumstances, representing an “other-oriented” emotional response; and the PD subscale assesses feelings of fear and discomfort when confronted with others’ suffering, reflecting a “self-oriented” response. The first two subscales quantify the cognitive dimension of empathy, namely the rational understanding of others’ mental states, whereas the latter two subscales capture the affective dimension of empathy, namely the self-referential emotional responses to others’ situations (Wang et al., 2021).

Following recruitment and the acquisition of informed consent, all questionnaires were completed before the MRI scanning session. Both the questionnaire assessments and MRI data acquisition took place between May 2022 and January 2023. Participants were required to complete the assessments independently in a quiet, well-lit room free from distractions.

### MRI data acquisition and preprocessing

Imaging data were acquired using a 3.0 Tesla MRI scanner (General Electric Discovery MR750; Milwaukee, WI, USA) equipped with a standard 8-channel phased-array head coil. To minimize scanner noise and head motion, participants were provided with earplugs and their heads were stabilized with foam padding. Resting-state fMRI data (blood-oxygen-level dependent (BOLD) signals) were collected using a gradient-echo echo-planar imaging (GRE-EPI) sequence. For dancers, musicians, and control participants, 32 axial slices were acquired with the following parameters: repetition time (TR) = 2,000 ms, echo time (TE) = 30 ms, flip angle (FA) = 90°, field of view (FOV) = 240 × 240 mm^2^, matrix = 64 × 64, and slice thickness = 4 mm. Each resting-state scan lasted 510 s, during which participants were instructed to keep their eyes closed, remain awake, and avoid head movement, resulting in 255 whole-brain volumes per subject.

Preprocessing of resting-state fMRI data was conducted using the standard pipeline implemented in the DPABI toolbox (http://rfmri.org/dpabi) (Yan et al., 2016). The procedure included: (1) conversion of raw DICOM data to NIFTI format; (2) removal of the first five volumes to account for scanner instability and participants’ adaptation to the environment; (3) slice-timing correction using the middle slice as the reference; (4) head motion correction, ensuring that translational movement did not exceed 1.5 mm and rotational movement did not exceed 1.5°; (5) spatial normalization to the MNI template with resampling to 3 × 3 × 3 mm^3^ voxels; (6) spatial smoothing with an 8-mm full-width-at-half-maximum (FWHM) Gaussian kernel; (7) nuisance regression including white matter signal, cerebrospinal fluid signal, Friston-24 head motion parameters (Friston et al., 1996), and volumes with framewise displacement (FD) > 0.2 as calculated by the Jenkinson method (Jenkinson et al., 2002; Power et al., 2012); and (8) temporal band-pass filtering (0.01–0.08 Hz) to reduce the effects of physiological noise and low-frequency drifts.

### Selection of brain network nodes

Based on previous studies, this research selected core regions of the Default Mode Network (DMN), Salience Network (SN), and Executive-Control Network (ECN) (Modi et al., 2015; Seeley et al., 2007; Sylvester et al., 2012), including a total of 27 brain regions as seed points. These three core networks were selected as they represent the fundamental functional architecture for empathy. Specifically, the DMN is primarily involved in self-referential thought and perspective-taking (Raichle, 2015), the SN serves to evaluate the salience of emotional stimuli (Seeley et al., 2007), and the ECN supports the higher-order cognitive control necessary for regulating empathic responses (Dosenbach et al., 2007).

Additionally, limbic MNS structures play a critical role in the generation of empathy. Based on relevant research findings (Cattaneo & Rizzolatti, 2009; Decety, 2011), this study further included four subregions of the amygdala and twelve regions of the basal ganglia from the Brainnetome (BN) Atlas as ROIs. In total, 41 brain regions were included in the network analysis, with two regions overlapping across networks. The mapping of representative functional clusters to the BN atlas is summarized in Table 2. These ROIs were used to construct brain networks in order to investigate the effects of dance and music training on the core empathy network.

**Table 2:** Mapping of identified functional clusters to the brainnetome (BN) atlas. Functional clusters identified in the study, their descriptive names, and their corresponding regions within the brainnetome Atlas along with their abbreviations. These regions served as the basis for defining the Regions of Interest (ROIs).

Cluster	Cluster name	Corresponding BN region	Abbr.
Cluster 1	Left anterior cingulate and paracingulate gyri	Left cingulate gyrus	CG_L_7_7
		Right orbital gyrus	OrG_R_6_1
		Left superior frontal gyrus	SFG_L_7_4
		Right superior frontal gyrus	SFG_R_7_4
Cluster 2	Right middle temporal gyrus	Right middle temporal gyrus	MTG_R_4_3
Cluster 3	Left lingual gyrus	Left cingulate gyrus	CG_L_7_4
Cluster 4	Left inferior frontal gyrus (triangular part)	Left inferior frontal gyrus	IFG_L_6_5
Cluster 5	Right middle frontal gyrus	Right middle frontal gyrus	MFG_R_7_1

### Within-network degree and between-network degree

Graph theory-based network analysis provides an effective theoretical framework and computational tool for exploring the intrinsic functional properties of the brain by abstracting it into a set of nodes and edges (Hwang et al., 2017). This approach enables quantitative characterization of network topology, thereby revealing the organisational principle of large-scale functional brain networks. Building on the concept of WMD (Guimerà & Amaral, 2005), the present study introduced two novel complementary graph-theoretical metrics, WND and BND, to assess connectivity pattern at the nodal and network levels.

A binary adjacency matrix was first constructed for each participant. Mean time series of 246 brain regions were extracted, and Pearson correlation coefficients were calculated between each pair of regions, resulting in a $\; 246 \times 246\;$ functional connectivity matrix. To eliminate weak connections and ensure that the results were not affected by a single threshold, a range of network density thresholds (0.10–0.15, increment = 0.001) was used to sparsify the positive correlation matrix and generate binary adjacency matrices at each density threshold. Subsequently, at each density threshold, the WND and BND were calculated for the ROIs defined in Selection of brain network nodes section. The formulas for WND and BND are formally similar to that for WMD, as shown in Formulas (1) and (2):

(1) $${Z_{iWND}} = {\rm \; }{{{K_{iS}} - \overline {{K_S}} } \over {{\sigma _{{K_S}}}}}$$

(2) $${Z_{iBND}} = {\rm \; }{{{K_{iS}} - \overline {{K_i}} } \over {{\sigma _{{K_i}}}}}$$

where ${K_{iS}}\;$ represents the number of edges between node i and all nodes in functional network S; $\overline {{K_S}} \;$ denotes the average number of edges among all nodes within network S; ${\sigma _{{K_S}}}\;$ is the standard deviation of the number of edges among nodes in S; $\overline {{K_i}} \;$ refers to the average number of edges between node i and all functional networks; and ${\sigma _{{K_i}}}\;$ is the standard deviation of edges between node i and all functional networks.

In general, nodes within the same functional network are assumed to exhibit stronger connectivity. WND reflects the standardized value of the connection strength between node i and network S relative to the overall connectivity strength within that network, whereas BND assesses the standardized connection strength of node i with network S relative to its connectivity with other networks. Finally, the results obtained across different thresholds were averaged.

### Statistical analysis and correlation analysis

First, a one-way analysis of variance (ANOVA) was performed to examine group differences in WND and BND among dancers, musicians, and controls, with age, sex, years of education, and mean framewise displacement included as covariates. Since both graph-theoretical metrics violated the assumption of normality, permutation testing (10,000 iterations) was employed to assess the significance of the F-values, with the threshold set at p<0.001. *Post hoc* analyses were further conducted for significant results. Second, partial correlation analyses were performed to evaluate the associations between WND, BND, and C-IRI subscale scores, while controlling for age, sex, years of education, and mean framewise displacement. The statistical significance threshold for these correlations was set at p<0.05.

## Results

### Comparison of within-network degree in the empathy network

As shown in Fig. 1, brain networks were constructed based on 246 ROIs, and a one-way ANOVA was performed to assess the WND of specific ROIs across dancers, musicians, and control participants. The results revealed significant group differences in the WND of the right medial amygdala with the subcortical network, the right nucleus accumbens and right dorsolateral putamen with the ventral attention network, the left superior frontal gyrus, right superior frontal gyrus, and left inferior frontal gyrus with the sensorimotor network, as well as the left cingulate gyrus with the limbic network.

**Figure 1:**
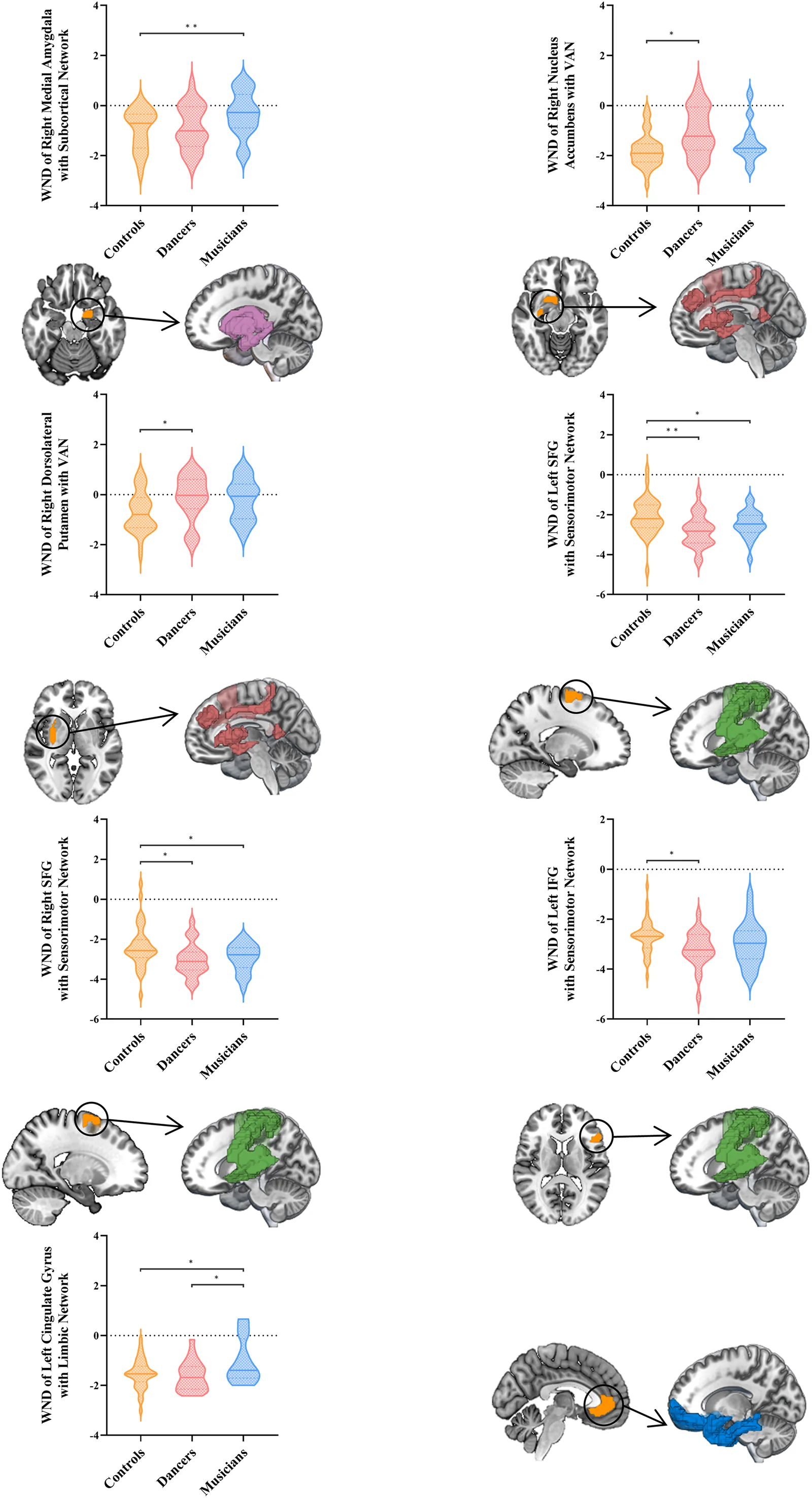
Results of ANOVA and *post hoc* tests for within-network degree of regions of interest. Group differences in within-network degree (WND) across dancers, musicians, and controls. Violin plots display the WND values for significant Regions of Interest (ROIs) across the three groups. Brain maps indicate the anatomical locations of these ROIs, including the right medial amygdala, right nucleus accumbens, right dorsolateral putamen, and frontal gyri. The internal box plots within the violins represent the median and interquartile ranges. Significant group differences were determined by ANOVA and *post hoc* permutation testing. Note: *p < 0.05, **p < 0.01.

*Post hoc* analyses of the ANOVA results further indicated the following:

(1) Dance-specific findings: Compared with the control group, dancers exhibited increased WND of the right nucleus accumbens and right dorsolateral putamen with the ventral attention network, while showing decreased WND of the left inferior frontal gyrus with the sensorimotor network.
(2) Music-specific findings: Compared with the control group, musicians exhibited increased WND of the right medial amygdala with the subcortical network and the left cingulate gyrus with the limbic network.
(3) Shared findings between dancers and musicians: Compared with the control group, both dancers and musicians exhibited decreased WND of the left superior frontal gyrus and right superior frontal gyrus with the sensorimotor network.

### Comparison of between-network degree in the empathy network

As shown in Fig. 2, the one-way ANOVA of BND across the three groups revealed significant differences in the right ventral caudate and right dorsolateral putamen with the ventral attention network, the right orbital gyrus with the frontoparietal network, the right superior temporal gyrus with the limbic network, and the right middle temporal gyrus with both the sensorimotor and subcortical networks.

**Figure 2:**
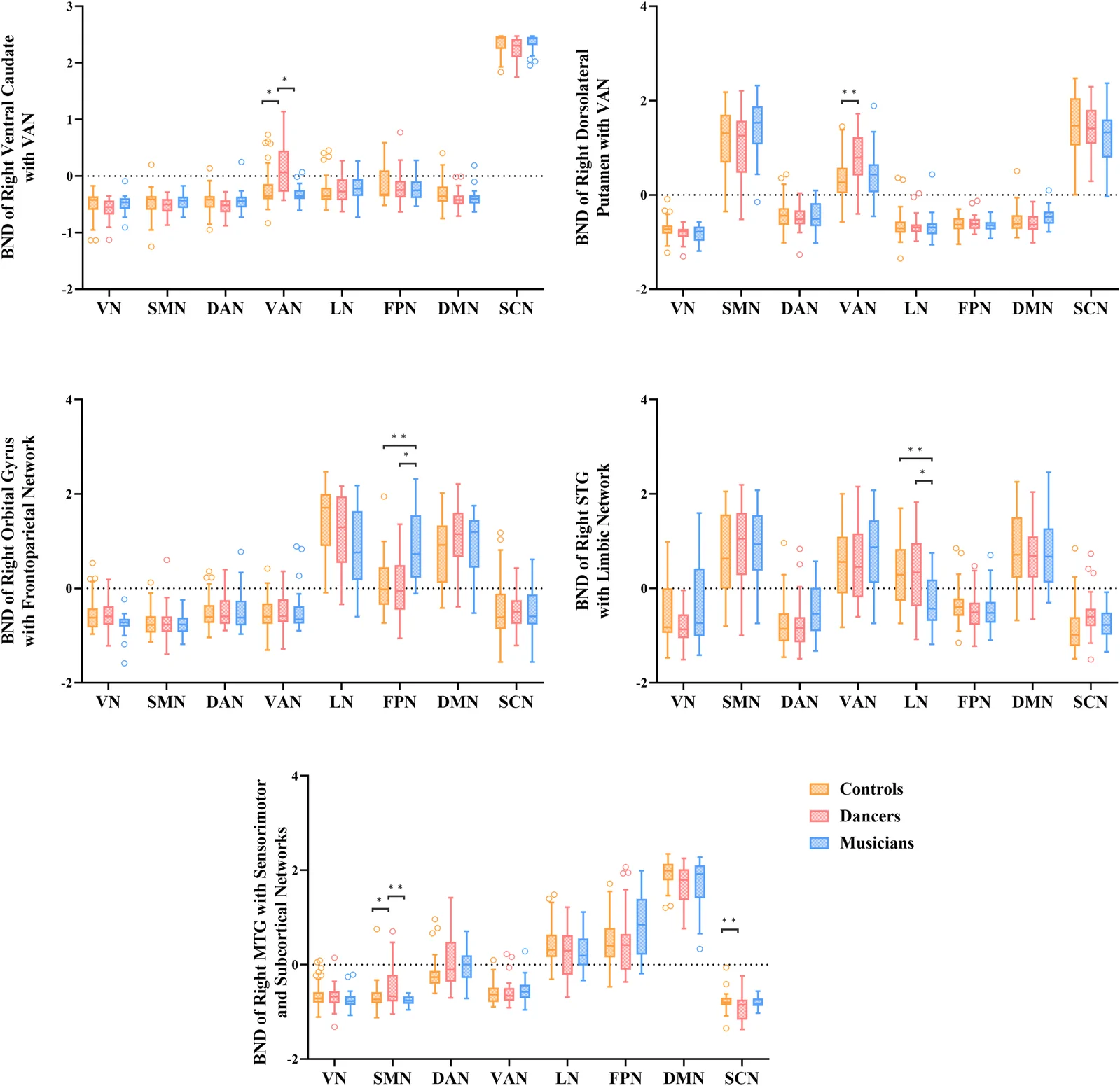
Results of ANOVA and *post hoc* tests for between-network degree of regions of interest. Group differences in between-network degree (BND) across the three groups. Box plots illustrate the BND values for specific ROIs interacting with target networks (*e.g*., Ventral Attention Network, Frontoparietal Network). Significant alterations were observed in the right ventral caudate, right dorsolateral putamen, right orbital gyrus, and right temporal gyrus. The colored boxes represent the interquartile range (IQR), and the whiskers extend to the minimum and maximum values. Note: *p < 0.05, **p < 0.01.

*Post hoc* analyses of the ANOVA results further indicated the following:

(1) Dance-specific findings: Compared with the control group, dancers exhibited increased BND of the right ventral caudate and right dorsolateral putamen with the ventral attention network, as well as increased BND of the right middle temporal gyrus with the sensorimotor network. In contrast, they showed decreased BND of the right middle temporal gyrus with the subcortical network.
(2) Music-specific findings: Compared with the control group, musicians exhibited increased BND of the right orbital gyrus with the frontoparietal network, while showing decreased BND of the right superior temporal gyrus with the limbic network.

### Correlation between within-network/between-network degree and empathy scale scores

As shown in Fig. 3, correlation analyses between WND and the subscales of the C-IRI revealed that, in the dance group, the WND values of the left cingulate gyrus and the limbic network were significantly negatively correlated with the Personal Distress (PD) subscale. In the music group, the Perspective Taking (PT) subscale showed a significant positive correlation with the WND values of the right medial amygdala and the subcortical network, but a significant negative correlation with the WND values of the left cingulate gyrus and the limbic network. In the control group, the WND values of the right medial amygdala and the subcortical network were significantly positively correlated with the FS subscale, whereas the WND values of the right superior frontal gyrus and the sensorimotor network were significantly negatively correlated with the total C-IRI score. Moreover, across all participants, the WND values of the right superior frontal gyrus and the sensorimotor network were significantly negatively correlated with the PD subscale.

**Figure 3:**
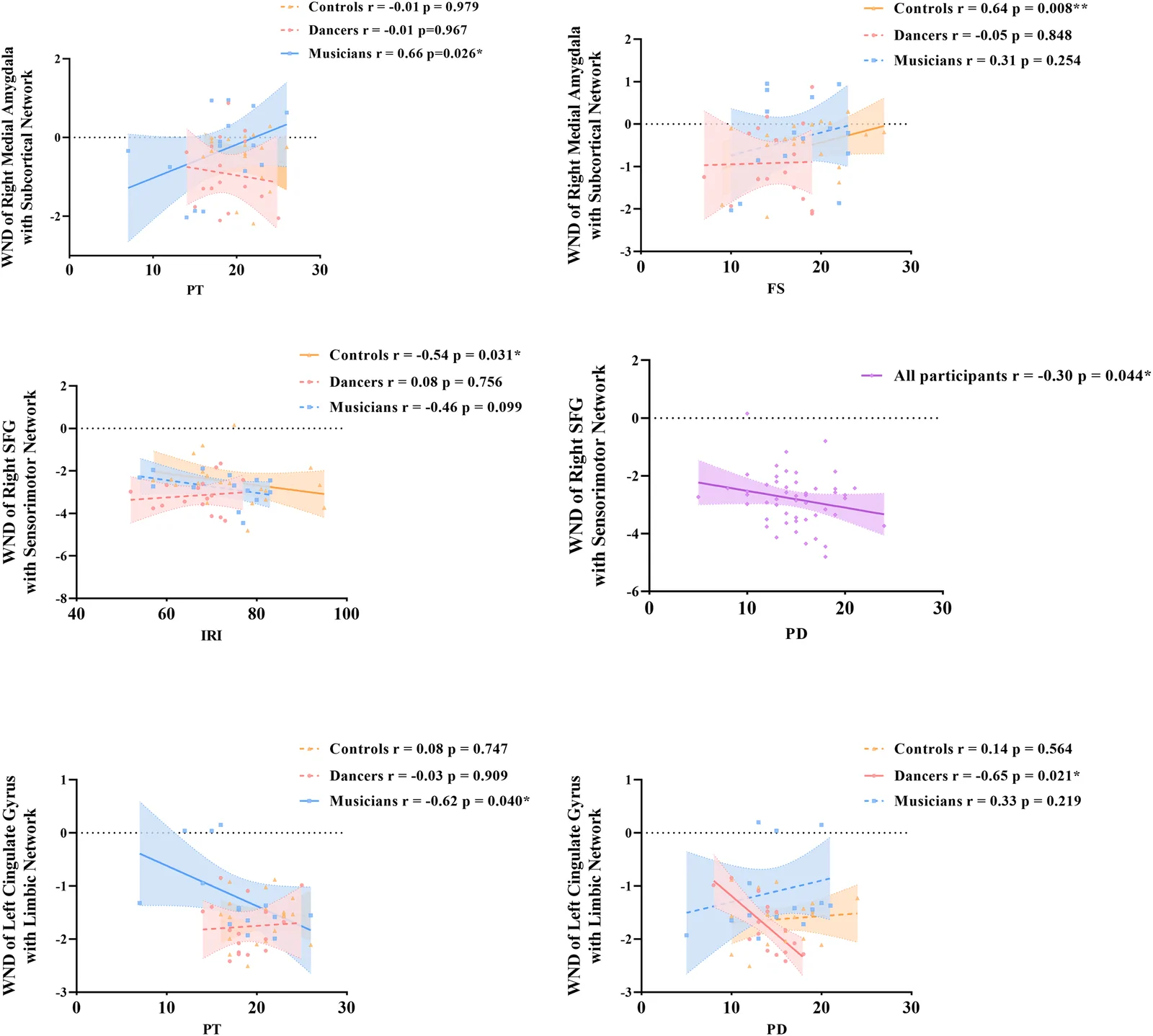
Correlation results between within-network degree and empathy scale scores. Correlation analyses between within-network degree (WND) and C-IRI empathy subscale scores. Scatter plots showing the linear relationship between WND values in specific brain regions and empathy scores (*e.g*., Personal Distress, Perspective Taking). Blue dots/lines represent the Control group, Pink represents Dancers, and Orange represents Musicians. Solid regression lines indicate statistically significant correlations (*p* < 0.05), while dashed lines indicate non-significant trends. The shaded areas represent the 95% confidence intervals.Note: *p < 0.05, **p < 0.01.

As shown in Fig. 4, correlation analyses between BND and the subscales of the C-IRI revealed that, in both the dance group and the control group, the BND values of the right ventral caudate and the ventral attention network were significantly positively correlated with the PD subscale. In the music group, the BND values of the right orbital gyrus and the frontoparietal network were significantly negatively correlated with the PD subscale. Moreover, across all participants, the BND values of the right middle temporal gyrus and the subcortical network were significantly positively correlated with the Empathic Concern (EC) subscale.

**Figure 4:**
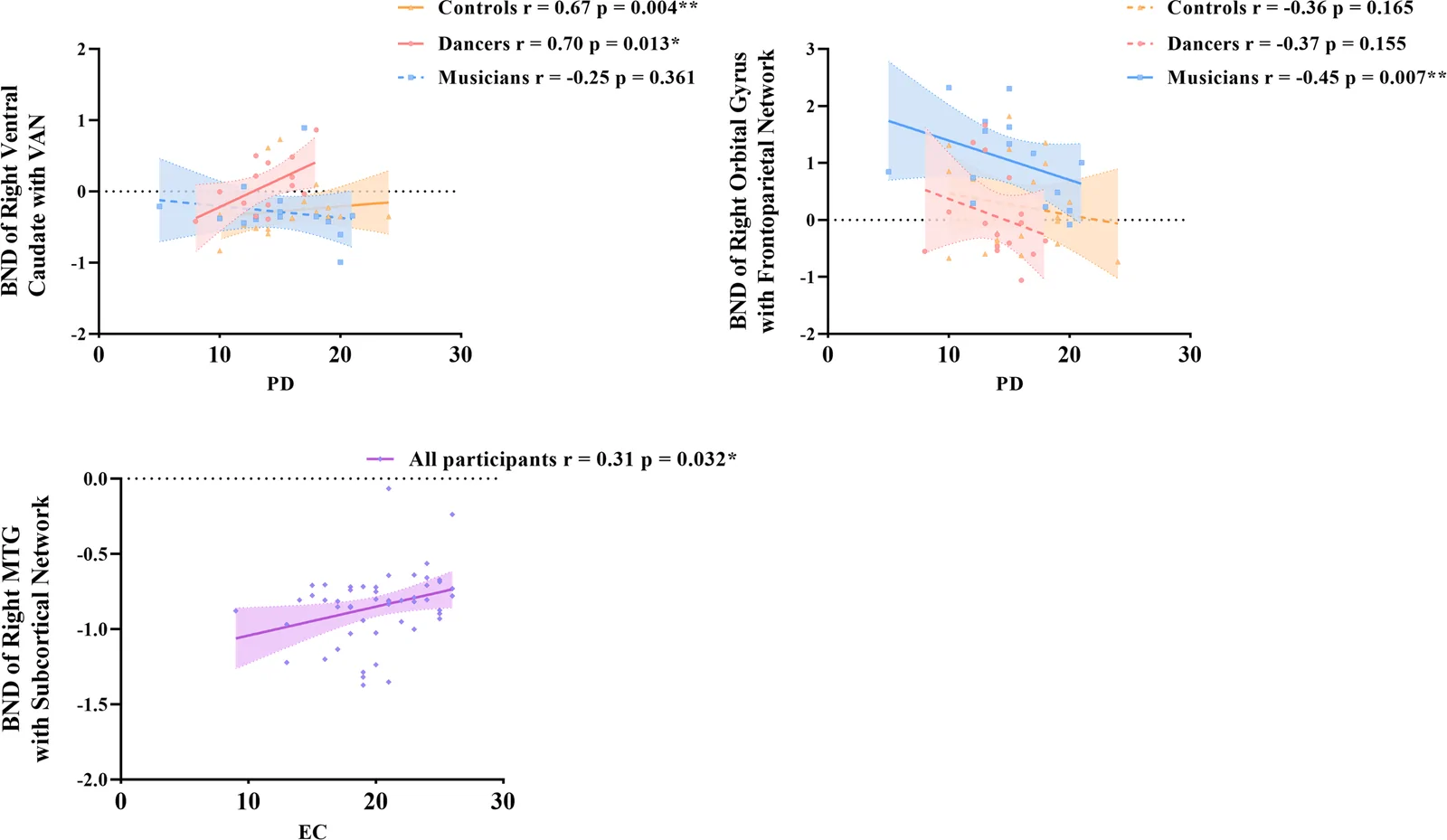
Correlation results between between-network degree and empathy scale scores. Correlation analyses between between-network degree (BND) and C-IRI empathy subscale scores. Scatter plots demonstrating the associations between inter-network connectivity strength (BND) and empathy dimensions (Personal Distress, Empathic Concern). Blue represents Controls, Pink represents Dancers, and Orange represents Musicians. Solid lines indicate significant correlations (*p* < 0.05), while dashed lines indicate non-significant correlations. Shaded areas indicate the 95% confidence intervals. Note: *p < 0.05, **p < 0.01.

## Discussion

To investigate the effects of dance and music training on the efficiency of cross-modular information transfer within the core empathy network, we used graph-theoretical analysis to examine connectivity patterns among core empathy-related brain regions and incorporated subcortical structures, specifically the amygdala and basal ganglia, as ROIs within large-scale brain networks. Two metrics, WND and BND were calculated to characterise intra- and inter-network connectivity. The results revealed that dance training enhanced the connectivity between the basal ganglia and the ventral attention network, whereas music training strengthened the connectivity between the amygdala and the subcortical network. Both types of training, however, were associated with reduced connectivity between the superior frontal gyrus and the sensorimotor network. Notably, these alterations were closely related to improvements in empathic ability.

### Different emphases of dance and music training in the empathy process

Empathic experience is an intersubjective process through which individuals understand others’ emotional states in everyday life (Innamorati et al., 2019). Its underlying neural processes can be broadly divided into three components: affective arousal, emotional perception and understanding, and emotion regulation (Decety, 2005; Decety & Meyer, 2008).

In the dance group, the WND values between the right nucleus accumbens, right dorsolateral putamen, and the ventral attention network were significantly increased, while the BND values between the right ventral caudate, right dorsolateral putamen, and the ventral attention network were also significantly enhanced. The nucleus accumbens integrates cognitive and affective information from the frontal and temporal lobes and is considered to be involved in emotional perception and affective understanding (Berridge & Kringelbach, 2015; Decety, 2011). Previous studies have demonstrated that the nucleus accumbens is activated not only by pleasant facial expressions but also by aversive stimuli (Ernst et al., 2004; Kelley, 2004; Reynolds & Berridge, 2002). Given that dance training requires continuous observation of others’ body language and facial expressions, it may enhance the dynamic encoding of emotional valence in the nucleus accumbens, thereby improving the accuracy of emotional perception. As a core node of the basal ganglia’s reward-motor loop, the putamen has been widely reported to be activated during emotional perception and induction tasks and is further associated with self-reported affective empathy (Phan et al., 2002, 2004). The enhanced functional connectivity between the putamen and the ventral attention network suggests that long-term dance training may promote synaptic myelination through reward-related feedback from movement, thereby improving the efficiency of attentional modulation by reward signals. The caudate nucleus is the key structure of reward prediction errors and plays a crucial role in emotion processing. There is evidence that the intensity of the activation in children’s caudate nucleus in response to fearful faces can predict their social skills, which highlights the key role in emotion perception (Hamilton & Alloy, 2016). The enhanced connectivity observed between the caudate nucleus and the ventral attention network may reflect the generation of predictive error signals, which adjust the allocation of attention resources by comparing the expected results and actual results during dance training. In addition, this enhanced connectivity was significantly positively correlated with the PD subscale, indicating that dance training enables dancers to quickly identify the intentions of others and dynamically adjust their attention, thus improving the accuracy of their empathy response.

The observed differences in functional connectivity between dancers and musicians may reflect specific training-induced neuroplasticity rather than inherent empathic traits. MNS activity is significantly modulated by associative sensorimotor learning and the acquisition of expertise (Heyes & Catmur, 2022). Consequently, the distinct connectivity patterns identified in this study could be attributed to the specialized demands of each discipline, specifically the gross-motor imitation required in dance *vs*. the fine auditory-motor coupling in music. Furthermore, the involvement of subcortical regions such as the amygdala and basal ganglia provides empirical support for the model in which affective empathy relies on visceromotor resonance (Bonini et al., 2022). These findings suggest that professional artistic training may modulate empathy by reshaping neural pathways responsible for both sensorimotor simulation and internal physiological representation.

Music training, on the other hand, specifically enhances the functional connectivity between the right medial amygdala and the subcortical network. The amygdala is a key neural component of emotional arousal and integrates external and interoceptive signals (Decety, 2010; Morris et al., 1998). Comparative studies between humans and monkeys show that amygdala damage can lead to a general reduction in emotional response (Aggleton, 1992). Repeated processing of auditory emotional cues during music training may strengthen communication between the amygdala and other subcortical regions, thereby enhancing emotional arousal. The positive association between this connectivity and cognitive empathy suggests that effective emotional arousal may provide essential physiological input for higher-order mentalizing processes.

In summary, dance training plays a crucial role in emotion perception, understanding and regulation, while music training mainly enhances emotional arousal in the process of empathy. Despite these differences, both dance and music training appear to share neural mechanisms that enhance the efficiency of empathic processing. Dance and music training are both forms of motor skill learning that require intensive movement imitation (Buccino et al., 2004). In the present study, both types of training were found to weaken the functional connectivity between the superior and inferior frontal gyri and the sensorimotor network, potentially reflecting neural pruning induced by sustained imitation. The inferior frontal gyrus (IFG) is responsible for decoding movement intentions and providing motor feedback during movement learning and imitation (Buccino et al., 2004). Previous studies have shown that dancers have significantly reduced functional connectivity between the IFG and multiple regions in the temporal, parietal, and sensorimotor cortices, potentially indicating a more efficient neural processing mechanism and less redundant connections (Lu et al., 2018). The superior frontal gyrus (SFG) is primarily involved in emotion regulation and play a key role in distinguishing self-related emotions from other-related emotions (Denny et al., 2012). Structural studies have further demonstrated a significant reduction in gray matter volume within the SFG of professional dancers, which may represent structural optimization resulting from long-term, high-intensity training (Hänggi et al., 2010). The observed reduction in frontal-sensorimotor connectivity in both dance and music training suggests that prolonged action imitation may promote synaptic pruning of redundant connections and establish more efficient information pathways, thereby optimizing brain function (Lu et al., 2018). Moreover, the negative correlation between this connectivity and empathy scale scores indicates that dance and music training may enhance synaptic pruning within self-referential pathways, reducing excessive self-monitoring of movement and freeing cognitive resources to support the development of affective empathy.

The present study found that only dance training enhanced the functional connectivity between the right middle temporal gyrus (MTG) and the sensorimotor network, which may result from improved auditory-perceptual-motor synchronization induced by dance practice. On the contrary, the reduction in connectivity between the right MTG and the subcortical network may reflect the optimization of cross-modal information routing, which leads to more specialized auditory cortex. The positive correlation between this connection mode and the EC subscale shows that reducing redundant emotional information transmission may help suppress excessive affective empathetic responses.

### Music training enhances cognitive abilities in a specific manner

This study further shows that the changes in emotional brain network connectivity induced by music training are negatively related to the improvement of empathy. Music, as an art form requiring structured and abstract thinking (Walton, 1988; Zaiets & Zaiets, 2022), may exert differential effect on cognitive empathy (Rot et al., 2025). Due to the wide variation of musical elements such as pitch, tone, rhythm, and melody, the player’s perception and interpretation of music is not only sound, but also a complex expression containing technical skills, cultural background and emotional intentions (Jackendoff & Lerdahl, 2006). Therefore, perceiving and understanding music requires reasoning about abstract musical elements, which in turn enhances rational and cognitive processing ability. Enhanced connectivity between the right orbitofrontal cortex (OFC) and the frontoparietal network in the music group may reflect top-down regulatory processes that dampen excessive emotional responses, thereby modulating emotional empathy. Strengthened connectivity between the left cingulate gyrus and the limbic network suggests improved cognitive control over emotional processing, potentially due to the need to maintain technical precision while expressing emotion during musical performance. The negative correlation between this connectivity and empathy scores aligns with prior findings that pianists show stronger anterior cingulate-amygdala connectivity, greater emotional control during improvisation, and lower trait empathy (Alluri et al., 2015). This shows that enhancing emotional inhibition and regulation may reduce self-reported empathy and improve empathy efficiency in professional or performance-related environments.

### Limitations

Although this study provides valuable insights, several limitations exist. First, the relatively small sample size and incomplete behavioral data necessitate viewing these findings as preliminary. Second, the cross-sectional design prevents causal inferences, requiring future longitudinal studies. Third, the reliance on self-report measures highlights the need for more objective assessments, such as behavioral tasks or biological indicators (Christensen, Gaigg & Calvo-Merino, 2018).

Furthermore, while our results highlight mechanisms in professional experts, clinical therapies predominantly target amateur participants and interpersonal dynamics. Future research should therefore investigate whether social ‘mirroring’ and partnered improvisation yield similar neuroplastic benefits in general populations. Additionally, to establish robust, evidence-based rehabilitation frameworks across diversified genres and cultural contexts, it is essential to explore neural activity during the training process itself and translate these findings into structured, standardized intervention protocols.

## Conclusions

Using graph-theoretical network analysis, this study applied WND and BND metrics to examine how dance and music training influence information transmission within the core empathy network. Both forms of training optimized connectivity between the superior frontal gyrus and the sensorimotor network, potentially freeing cognitive resources to support enhanced empathy. Dance training specifically strengthened communication between the basal ganglia and the ventral attention network, while music training enhanced connectivity between the amygdala and the subcortical network, supporting affective and cognitive empathy through more efficient emotional arousal and regulation. By characterizing the topological features of the core empathy network from a cross-network integration perspective, the present findings offer a theoretical foundation for developing art-based intervention programs.

## Supplemental Information

10.7717/peerj.21811/supp-1 Supplemental Information 1 Individual functional connectivity (FC) matrices (246 regions) for the Musician and Dancer groups.

10.7717/peerj.21811/supp-2 Supplemental Information 2 Individual FC matrices for the Control group. the “ROI” folder (NIfTI files for the core empathy network), and the calculated result files (*.mat) for Within-Network Degree (WND) and Between-Network Degree (BND).

10.7717/peerj.21811/supp-3 Supplemental Information 3 Output Figures. raw figure outputs generated automatically by the MATLAB scripts.

10.7717/peerj.21811/supp-4 Supplemental Information 4 Code plot bar posthoc. MATLAB Code: MATLAB script used to generate bar plots for visualizing the results of the *post-hoc* analyses (*e.g*., group-wise comparisons) after the main ANOVA was performed. These plots aid in the interpretation of the significant group differences found.

10.7717/peerj.21811/supp-5 Supplemental Information 5 Code permutationBND network. MATLAB Code: MATLAB script to perform the one-way ANOVA (*via* permutation testing) on the Between-Network Degree (BND) values to assess group differences. The statistical results generated by this script correspond to Figure 2 in the manuscript.

10.7717/peerj.21811/supp-6 Supplemental Information 6 Code permutationWMD within. MATLAB Code: MATLAB script to perform the one-way ANOVA (*via* permutation testing) on the Within-Network Degree (WND) values to assess group differences between dancers, musicians, and controls. The statistical results generated by this script correspond to Figure 1 in the manuscript.

10.7717/peerj.21811/supp-7 Supplemental Information 7 Code plot behavior WMD. MATLAB Code: MATLAB script for performing the correlation analysis between the graph-theoretical metrics (WND and BND) and the behavioral empathy scale scores (C-IRI). This script is also used to generate the corresponding scatter plots shown in Figures 3 and 4.

10.7717/peerj.21811/supp-8 Supplemental Information 8 Code WND&BND. MATLAB Code: MATLAB script to calculate the core graph-theoretical metrics of the study: Within-Network Degree (WND) and Between-Network Degree (BND). This script takes the individual functional connectivity (FC) matrices as input and outputs the WND and BND values for each specified Region of Interest (ROI).

10.7717/peerj.21811/supp-9 Supplemental Information 9 Code WMD simplettest. MATLAB Code: A supplementary MATLAB script for conducting basic statistical t-tests on the calculated WND and BND metrics. This script was primarily used for exploratory analyses or simple group comparisons not detailed in the main manuscript figures.

## Additional Information and Declarations

### Data Availability

The following information was supplied regarding data availability:

The raw data, code, and output figures are available in the Supplemental Files.
